# The Involvement of Atlastin in Dengue Virus and *Wolbachia* Infection in Aedes aegypti and Its Regulation by aae-miR-989

**DOI:** 10.1128/spectrum.02258-22

**Published:** 2022-09-27

**Authors:** Mazhar Hussain, Twyla Bradshaw, Morris Lee, Sassan Asgari

**Affiliations:** a Australian Infectious Disease Research Centre, School of Biological Sciences, The University of Queensland, Brisbane, Australia; Center for Research and Advanced Studies (CINVESTAV-IPN)

**Keywords:** *Aedes aegypti*, dengue virus, atlastin, endoplasmic reticulum, microRNA, *Wolbachia*, mosquito

## Abstract

Endoplasmic reticulum (ER)-shaping atlastin proteins (ATLs) have been demonstrated to play a functional role during flavivirus replication in mammalian cells. For dengue virus (DENV), atlastin is required in the formation of the replication organelles and RNA replication, virion assembly, production of the infectious virus particles, and trafficking or directing the association of vesicle packets with furin. Here, we investigated the involvement of atlastin in DENV replication in the mosquito Aedes aegypti and explored the possibility of its manipulation by the endosymbiotic bacterium *Wolbachia* to interfere with DENV replication. Results showed the expression of *Ae. aegypti atlastin* gene (AaATL) was upregulated in DENV-infected Aag2 cells, and its silencing led to reduced DENV replication. Contrary to our assumption that AaATL could be downregulated by *Wolbachia*, we did not find evidence for that in *Wolbachia*-infected cell lines, but this was the case in mosquitoes. Further, silencing AaATL did not have any effect on *Wolbachia* density. Our results also suggest that aae-miR-989 miRNA negatively regulates AaATL. The oversupply of the miRNA mimic led to reduced DENV replication consistent with the positive role of AaATL in DENV replication. Overall, the results favor AaATL’s involvement in DENV replication; however, there is no support that the protein is involved in *Wolbachia*-mediated DENV inhibition. In addition, the results contribute to discerning further possible overlapping functions of ATLs in mosquitoes and mammalian cells.

**IMPORTANCE** Atlastin is a protein associated with the endoplasmic reticulum and has been shown to play a role in replication of flaviviruses in mammalian cells. This study aimed to investigate the role of mosquito Aedes aegypti atlastin (AaATL) in dengue virus replication and maintenance of *Wolbachia*, an endosymbiotic bacterium, in the mosquito. Our results suggest that AaATL facilitates dengue virus replication in mosquito cells, considering silencing the gene led to reductions in virus replication and virion production. Further, AaATL was found to be regulated by a mosquito microRNA, aae-miR-989. Despite an effect on dengue virus, AaATL silencing did not affect *Wolbachia* replication and maintenance in mosquito cells. The results shed light on the role of atlastins in mosquito-pathogen interactions and their overlapping roles in mosquito and mammalian cells.

## INTRODUCTION

Dengue virus (DENV) is transmitted by the primary mosquito vector Aedes aegypti that inhabits tropical and subtropical regions and is the leading cause of arthropod-borne viral diseases globally (1). Annually, there are about 390 million DENV infections, and 96 million of them are severe (2). DENV has an RNA genome of 10,700 nucleotides that includes 5′- and 3′- untranslated regions and a single open reading frame that encodes a single polyprotein (3). This polyprotein is cleaved into three structural proteins (capsid, premembrane/membrane, and envelope) and several nonstructural proteins, which are involved in viral RNA replication, virus assembly, and alteration of the host cell responses (4). Replication of DENV and other flaviviruses occurs on virus-induced host cell membranes and requires autophagy for efficient replication (3). There are four serotypes of DENV (DENV 1 to 4), each with genotype variability, and infection by one serotype results in exclusive long-term immunity to that serotype but not the others (5). Clinical symptoms of DENV in humans include dengue hemorrhagic fever/shock syndrome (DHS/DSS), severe muscle spasms and joint pain, and although most cases are acute, severe illness and death may occur (3).

Vaccine development for DENV has been complicated due to serotype variability (6). The E protein, responsible for viral cell entry, has been the major target of vaccine development; however, E protein neutralization and protection across the four DENV serotypes has been limited (7). Upon human infection with one serotype, heterologous protection is short term, and previous infection with one serotype can predispose to DHS/DSS. Therefore, in areas of endemicity where more than one DENV serotype is circulating, a dengue vaccine faces the obstacles of risking an induced immunological condition, enhanced disease, and fleeting immunity (7). In the absence of an effective vaccine to protect against all the four serotypes or an antiviral drug, dengue disease control has centered around vector control or reduction in viral transmission using nonchemical approaches. One such approach is utilization of *Wolbachia* as a biological control agent (8).

*Wolbachia* is a commonly found endosymbiotic bacterium in insects that depends on host nutrients such as amino acids and lipids, although it can be a nutritional symbiont (9, 10). It is mostly known for reproductive manipulations of the host, but *Wolbachia* has also been shown to stop replication of a variety of RNA viruses in *Drosophila* and mosquitoes; however, it is naturally less common in *Ae. aegypti* (10). Consequently, *Ae. aegypti* mosquitoes have been transinfected with a number of different strains of *Wolbachia* for population replacement with pathogen blocking property, and population suppression (8). However, the mechanism of pathogen blocking is poorly understood. The presence of *Wolbachia* induces changes in host gene expression and notable differences have been seen in antioxidant processes, metabolism, immune responses, and microRNAs (reviewed in references 11). In addition, *Wolbachia* competes with cytoplasmic replicating positive sense RNA viruses, such as DENV, for resources (11–13) and space (14). Therefore, a *Wolbachia*-modified host cellular environment is less favorable for cytoplasmic replicating viruses, such as DENV (15).

The ER is the largest cellular organelle and functions in many cellular processes that are assimilated by flaviviruses once infected (16). It is composed of several subdomains, including perinuclear membrane sheets and tubule-like structures that branch through the cell periphery (17). Virus particles bud into the ER lumen upon entry into the cell and are translated at the rough ER. The expressed DENV proteins can induce rearrangement of ER membranes into three distinct structures in order to develop a suitable environment for viral replication (17, 18). These distinct structures include vesicle packets (VPs), which are suspected to be the site of viral replication and therefore, regarded as the viral replication organelle (RO). The other two rearrangements are convoluted membranes (CM), and membrane vesicles (VE) (17, 18).

In *Wolbachia*-infected cells, it has been indicated that the ER could potentially be a source of vacuolar membrane and the interaction between *Wolbachia* cells (9). This particular organelle and derived intracellular vesicular trafficking play an important role in immune escape and control of apoptosis (9). Moreover, it has been shown that *Wolbachia* causes enhancement and redistribution of the ER at the subcellular level that results in highly enriched cytoplasmic regions of the tubular ER. Concurrently, a significant fraction of the ER contracts to become heavily clustered close to the nucleus (9).

The ER subdomain of tubule-like structure extends through the cell periphery, and its morphology is maintained by distinct membrane shaping and fusion proteins (17). Such fusion proteins include the ER resident membrane-bound GTPases and atlastins (ATLs) (17, 19). ATLs are required to produce ER tubules *in vitro*, and their dysfunction leads to formation of long, unbranched ER tubules, and mutated ATLs have been associated with neurodegenerative diseases (17, 19). These ER-shaping responses suggest that ATLs are ER fusogens, which maintain branched ER tubule networks, key cellular factors in regulating ER function, and important factors in human disease (17, 19). ATLs in mammalian cells have been shown to be host factors exploited by both DENV and Zika virus (ZIKV), potentially influencing the DENV assembly or production of infectious particles and trafficking/directing the association of VPs with furin (17, 19).

Considering the association of DENV and *Wolbachia* with the ER, we aimed to investigate the involvement of ATL in DENV and *Wolbachia* replication in the mosquito *Ae. aegypti*. While previous studies have indicated functional roles of ATLs in mammalian cells infected with DENV, to our knowledge, ATL has not yet been characterized for *Ae. aegypti* cells or challenged with *Wolbachia* and DENV. Previous studies of ATLs in mammalian cells determined that the proteins are required for the formation of the viral ROs and RNA replication, production of the infectious virus particles, and trafficking or directing the association of VPs with furin (17, 19). Moreover, current research has not yet deciphered the full mechanism(s) involved in *Wolbachia*’s virus-blocking phenotype. Therefore, we aimed to test the hypothesis that *Ae. aegypti* ATL (AaATL) has an involvement in flavivirus replication as seen in mammalian cells, and DENV replication is hindered upon *Wolbachia* infection because AaATL expression could be downregulated in the presence of *Wolbachia*. Further, we explored regulation of AaATL expression by a mosquito miRNA, aae-miR-989-3p. miRNA-target prediction suggested that among *Ae. aegypti* miRNAs aae-miR-989-3p has the highest potential to target AaATL. Further, this miRNA has been shown to be differentially expressed in response to *Wolbachia* and DENV infection in mosquitoes (20, 21).

## RESULTS

### Induction of AaATL expression in DENV-infected Aag2 cells.

When we searched the genome of *Ae. aegypti*, we could only find one atlastin gene (AaATL; NCBI XP_001663157.1, VectorBase AAEL003109), unlike human genome encoding three ATLs (ATL1-3); AaATL showed 57% and 59% amino acid sequence identities with HsATL2 and HsATL3, respectively (Fig. S1). Further, similar to HsATLs, AaATL contains a guanylate-binding domain (Fig. S1, the underlined region, aa 37 to 287).

**FIG 1:**
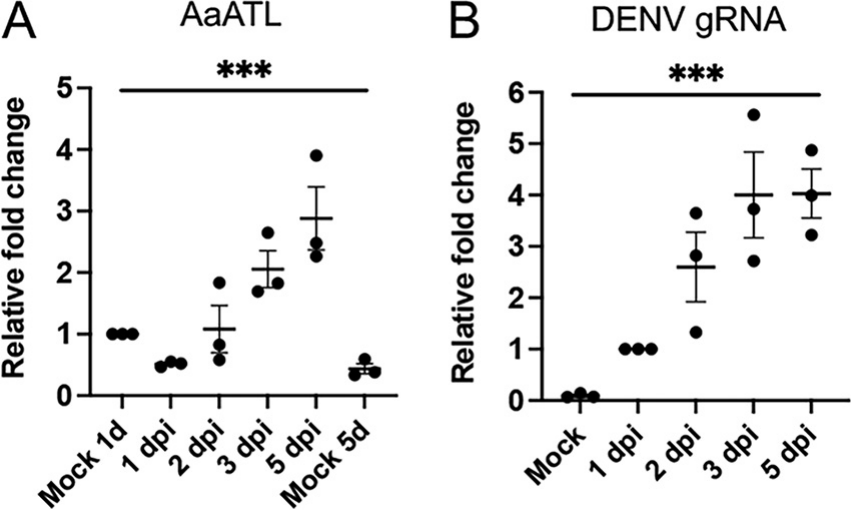
Expression of AaATL following DENV infection in Aag2 cells. (A) RT-qPCR analysis of the expression of AaATL at various time points following DENV infection of Aag2 cells. Noninfected cells (Mock) were collected alongside 1 and 5 dpi and used as negative controls. (B) Confirmation of DENV infection of Aag2 cells used in A. Uninfected cells (mock) were collected alongside 1 dpi and used as negative control. One-way ANOVA was used for statistical analysis. The error bars represent standard error of mean (SEM) of three biological replicates. Asterisks indicate significant difference between compared samples. ***, *P < *0.0001.

To assess the dynamics of the expression of AaATL upon DENV infection, Aag2 cells were infected with 1 MOI DENV and collected at 1 to 5 dpi. RT-qPCR analysis of RNA collected from cells showed gradual increase in AaATL transcript levels over time (Fig. 1A). The expression of AaATL at 5 dpi in comparison to that seen in mock-infected cells at 1 and 5 days was significantly higher (*P = *0.0004), suggesting that AaATL could be involved during DENV replication in Aag2 cells.

To ascertain DENV replication in the experiment, genomic RNA (gRNA) replication was assessed by RT-qPCR. As expected, mock cells showed no DENV replication. Over time, DENV replication could be seen to increase steadily (*P = *0.001) following the days postinoculation and then plateaued at 5 dpi (Fig. 1B). The results suggested that Aag2 cells were successfully infected with DENV.

### Silencing AaATL by RNAi and its effect on DENV replication.

**FIG 2:**
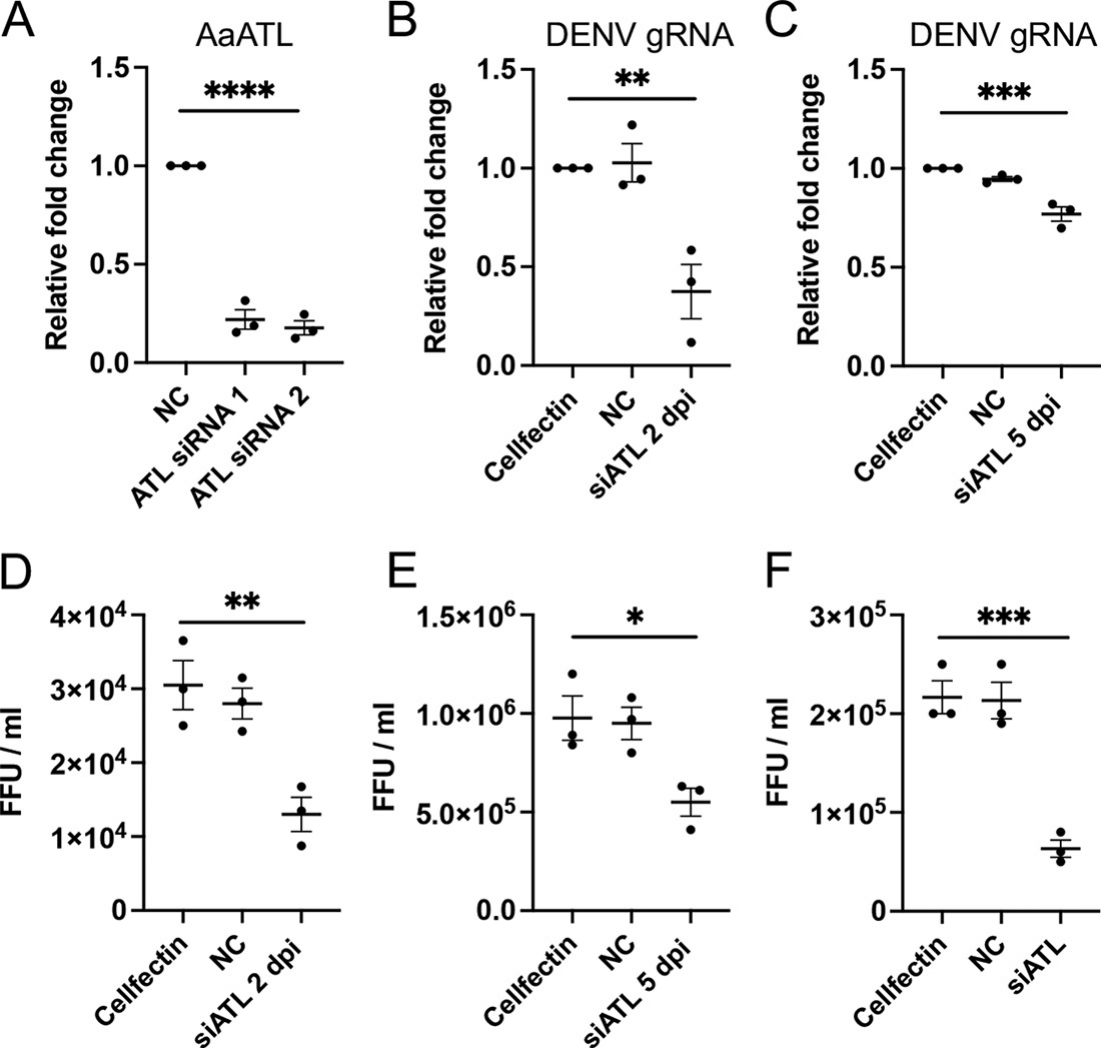
Silencing AaATL by siRNAs and its effect on DENV replication. (A) RT-qPCR analysis of RNA collected from Aag2 cells transfected with AaATL siRNA 1 and 2. NC, negative-control siRNA. (B) and (C) AaATL siRNA-transfected and DENV-infected Aag2 cells at 2 dpi and 5 dpi, respectively. Cellfectin and control siRNA (NC) were used as controls. (D) and (E) Supernatants collected from experiment in (B) and (C) were subjected to focus forming assay to quantify DENV virions. (F) Focus forming assay of DENV virions produced from AaATL silenced C6/36 cells transfected with AaATL siRNA for 2 days and infected with DENV for 3 days. FFU, focus forming units. One-way ANOVA test was carried out to determine statistical significance among groups. The error bars represent standard error of mean (SEM) of three biological replicates. Asterisks indicate significant difference between compared samples. *, *P < *0.05; **, *P < *0.01; ***, *P < *0.0001; ****, *P < *0.0001.

To silence AaATL, two synthetic siRNAs were used in transfection of Aag2 cells. AaATL expression was measured upon silencing the gene in transfected DENV-infected Aag2 cells (4 dpi). A very significant reduction (*P = *0.0001) was observed in the expression of AaATL in cells transfected with AaATL siRNA 1 and 2 (Fig. 2A; 78% and 82% average reductions, respectively) compared to the control siRNA assessed through RT-qPCR. The result confirmed that AaATL was successfully silenced by both siRNAs. Considering both siRNAs were effective in silencing AaATL, for further experiments, we only used siRNA 1 (ATL siRNA).

DENV genomic RNA replication was assessed at 2 and 5 dpi, after silencing AaATL in Aag2 cells with ATL siRNA to determine if replication of DENV was affected by atlastin expression. The RT-qPCR results showed significant reductions in DENV replication when AaATL was silenced at 2 dpi (*P = *0.0051; Fig. 2B) and 5 dpi (*P = *0.0008; Fig. 2C). Subsequently, focus-forming assay for DENV-2 virion titration was performed to verify the results of AaATL RNAi on DENV gRNA replication. Consistent with the gRNA results, at 2 and 5 dpi there were significant decreases (*P = *0.0067 and *P = *0.0265, respectively) in DENV virion titers (Fig. 2D and E). Further, silencing of AaATL in C6/36 cells, derived from *Ae. albopictus*, with siRNA led to significant reductions (*P = *0.0006) in DENV virion production assessed by focus forming assay 3 dpi postinfection (Fig. 2F). AaATL and *Ae. albopictus* ATL are 90% identical at the nucleotide level with only one mismatch at the ATL siRNA site (Fig. S2). Overall, the results suggest that ATL could be involved in DENV replication in mosquito cells.

### AaATL expression in *Wolbachia*-transinfected cells and mosquitoes and the effect of its silencing on *Wolbachia* density.

**FIG 3:**
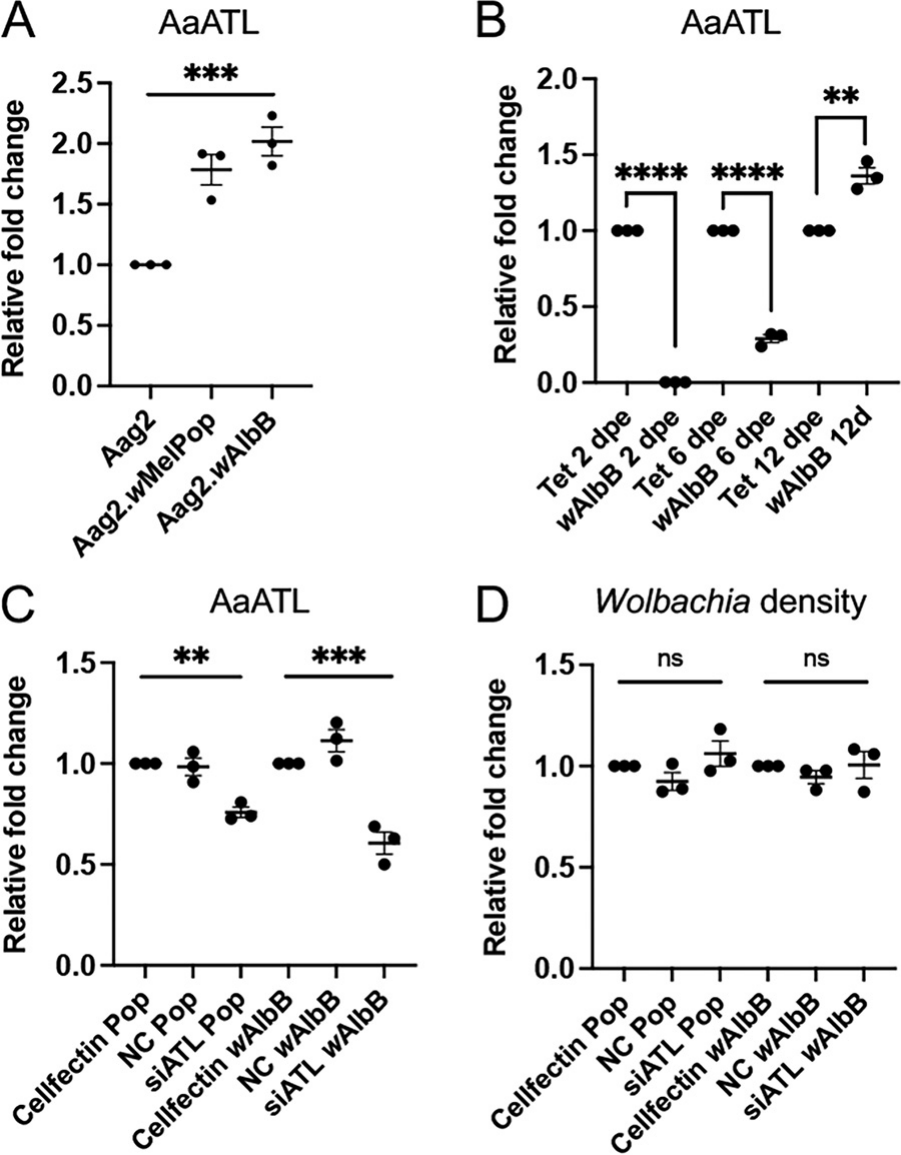
Expression of AaATL in *Wolbachia*-transinfected Aag2 cells and *Ae. aegypti* mosquitoes and the effect of its silencing on *Wolbachia*. (A) The expression levels of *AaATL* in Aag2.*w*AlbB and Aag2.*w*MelPop cells in comparison to *Wolbachia*-free Aag2 cells. (B) The AaATL expression comparison between *w*AlbB-transinfected and tetracycline-cured *Ae. aegypti* mosquitoes at 2, 6, and 12 days post emergence (dpe) assessed by RT-qPCR. (C) RT-qPCR analysis of RNA collected from Aag2.*w*MelPop and Aag2.*w*AlbB cells transfected with AaATL siRNA, negative control (NC), or Cellfectin transfection reagent. (D) qPCR analysis of DNA extracted from Aag2.*w*MelPop and Aag2.*w*AlbB cells treated as in C with siRNAs using specific primers to *RPS17* and *WSP* genes. The error bars represent standard error of mean (SEM) of three biological replicates. One-way ANOVA test was carried out to determine statistical significance among groups in A, C, and D. *t* test was used for statistical analysis in B. Asterisks indicate significant difference between compared samples. ns, not significant; **, *P < *0.01; ***, *P < *0.001; ****, *P < *0.0001.

Since previous studies have shown that *Wolbachia* blocks DENV replication in mosquito cells, we investigated whether *Wolbachia* could reduce AaATL expression and consequently restrict DENV replication. For this, AaATL expression levels were assessed in two Aag2 cell lines persistently infected with two different strains of *Wolbachia*, *w*AlbB, and *w*MelPop. The expression levels were compared with that of the Aag2 uninfected cells. RT-qPCR analysis of RNA extracted from the cells suggested significantly higher AaATL transcript levels (*P = *0.0008) in Aag2.*w*AlbB and Aag2.*w*MelPop cells compared to Aag2 cells (Fig. 3A).

The effect of *Wolbachia* on AaATL expression was further evaluated in *w*AlbB-transinfected and tetracycline-cured female *Ae. aegypti* mosquitoes at various days post emergence (2 dpe, 6 dpe, 12 dpe). When the expression levels of AaATL between *w*AlbB and tetracycline-cured *Ae. aegypti* mosquitoes were compared by RT-qPCR, significantly less AaATL transcript levels were found in *Wolbachia*-infected mosquitoes at 2 dpe and 6 dpe (Fig. 3B). However, at 12 dpe, there were significantly higher AaATL transcript levels in *Wolbachia*-infected mosquitoes. These results in mosquitoes are somewhat contradictory to those seen in the cell lines (Fig. 3A) and suggest that change in AaATL expression could be age and/or tissue/cell specific. For example, while at 12 dpe there is more AaATL expression in *Wolbachia*-infected mosquitoes compared to *Wolbachia*-free mosquitoes, consistent with cell line results, at 2 and 6 dpi, there is less AaATL expression suggesting age-related regulation of AaATL. Aag2 cells are rather homogenous cells, whereas mosquitoes are comprised of different cell types and tissues that may have differential expression levels of AaATL.

The density of *Wolbachia* was assessed upon RNAi of AaATL to evaluate whether the gene was required for *Wolbachia* maintenance in Aag2.*w*AlbB and Aag2.*w*MelPop cells. Silencing of AaATL was confirmed in the cells (Fig. 3C), however, there was no effect on *Wolbachia* density in either of the treated cells (Fig. 3D).

### aae-miR-989 regulates AaATL.

**FIG 4:**
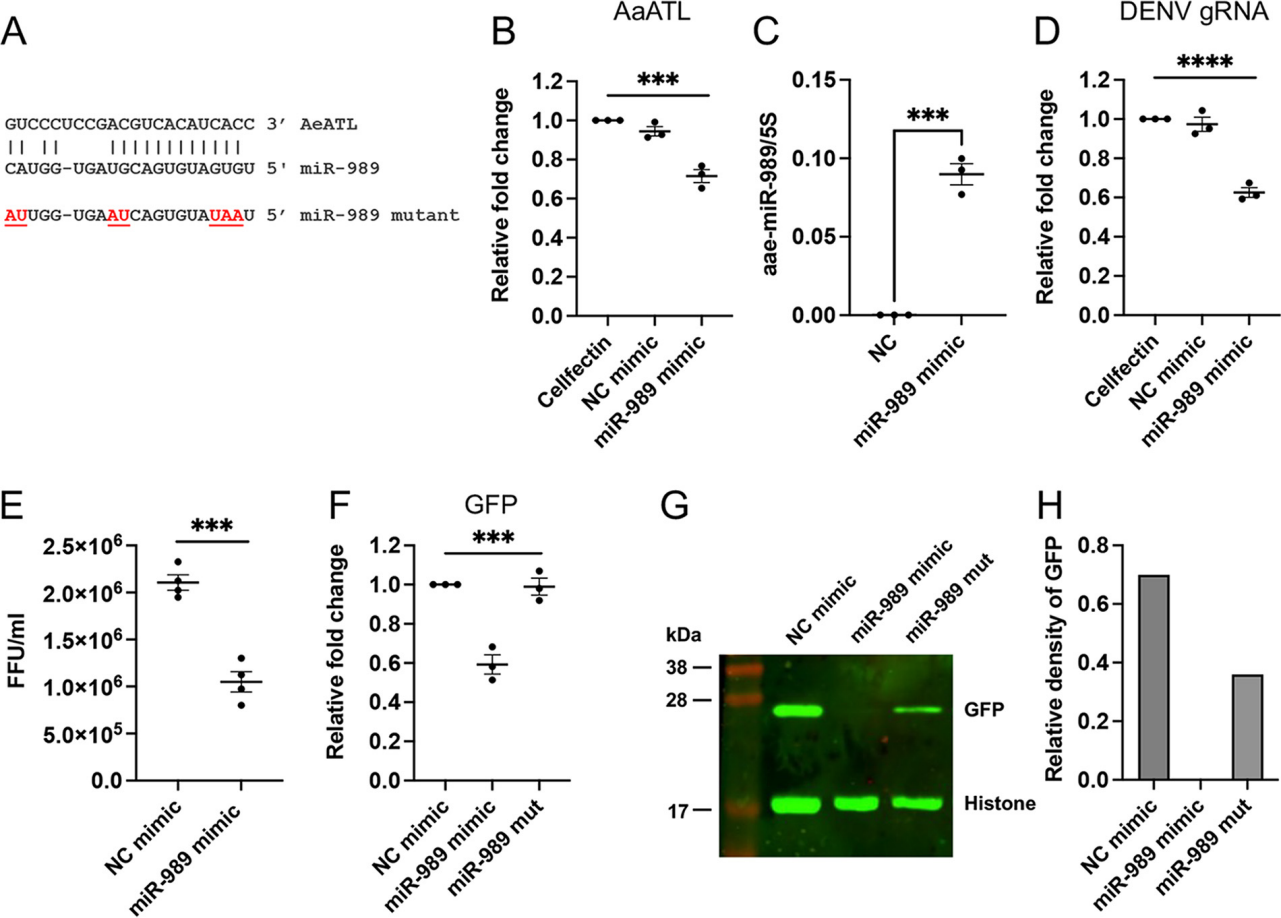
AaATL is regulated by aae-miR-989. (A) Complementary sequences of aae-miR-989 (miR-989) and AaATL. Sequences of a mutant synthetic mimic synthesized for follow-up experiments is shown (red nucleotides show mutated residues). (B) Effect of aae-miR-989 on AaATL expression was assessed by RT-qPCR analysis of RNA from Aag2 cells 5 days after transfection with the miRNA mimic, negative control (NC) mimic or Cellfectin transfection reagent only. (C) Confirmation of increase in the abundance of aae-miR-989 by RT-qPCR in mimic-transfected cells compared to NC-transfected cells. *t* test was used to determine statistical significance. (D) Effect of aae-miR-989 on DENV genomic RNA (gRNA) levels was assessed by RT-qPCR analysis of RNA extracted from Aag2 cells treated as in (B) and infected with 1 MOI DENV for 5 days. (E) Titration of DENV virions in the supernatants collected from a replicate of the experiment in D. FFU, focus forming unit. *t* test was used to determine statistical significance. (F and G) Validation of aae-miR-989 AaATL interaction. Sf9 cells were cotransfected with pIZ/GFP vector containing the target sequences of AaATL downstream of the *GFP* gene and aae-miR-989 mimic, mutant mimic (shown in panel A), NC mimic or Cellfectin only. RNA was extracted from the cells 3 days posttransfection and *GFP* transcript levels were assessed by RT-qPCR using primers to *GFP*, and *actin* as the normalizing gene (F) and protein samples from the same experiment analyzed on Western blot using antibodies to GFP and histone as control (G). (H) The relative density of GFP to histone in (G) determined by densitometric analysis using ImageJ. The error bars represent standard error of mean (SEM) of three biological replicates. One-way ANOVA test was carried out to determine statistical significance, expect for (C and E). ***, *P < *0.001; ****, *P < *0.0001.

Expression of host miRNAs have been reported to be altered by flaviviruses infection (22, 23). For example, in Culex quinquefasciatus, miR-989 and miR-92 were differentially expressed in response to West Nile virus infection (24). In *Ae. aegypti*, 35 miRNAs were differentially expressed upon DENV-2 infection, four of which were upregulated and the rest downregulated (22). With the upregulation of AaATL over the course of DENV infection, we hypothesized that AaATL could be regulated by miRNAs. To investigate this, all the *Ae. aegypti* miRNAs were screened for potential binding sites to AaATL using RNA22. One of the top candidates turned out to be aae-miR-989 with very good seed region complementarity and minimum free energy of −22.10 kcal/mol (Fig. 4A). This target sequence is also conserved in *Ae. albopictus* atlastin, except the first nucleotide from the 5′ end (G/A). In Aag2 cells 5 dpi, we found high levels of AaATL transcript levels Fig. 1A. To find out if aae-miR-989 has any effect on AaATL transcript levels, Aag2 cells were transfected with aae-miR-989 mimic, and 24 h after transfection they were infected with DENV. Five dpi, there was a significant reduction (about 30%; *P = *0.0011) in AaATL transcript levels compared to the controls transfected with negative-control mimic or the Cellfectin transfection reagent only (Fig. 4B). Increase in aae-miR-989 levels in mimic-transfected cells relative to NC was confirmed in the same samples (Fig. 4C). This suggested that the miRNA might negatively regulate AaATL expression. Next, replication of DENV in these cells was assessed. RT-qPCR results showed significant reduction (*P* = <0.0001) in DENV replication assessed by quantification of the viral gRNA (Fig. 4D). The titers of DENV virions also significantly declined (*P = *0.0002) in the presence of aae-miR-989 mimic (Fig. 4E). This is consistent with the previous results that AaATL is involved in DENV replication; since aae-miR-989 reduces AaATL expression, in turn, this reduction has a negative effect on DENV replication.

To further validate the sequence-specific interaction of aae-miR-989 with AaATL, the target sequence of AaATL was cloned downstream of the coding region for *GFP* in the pIZ/V5 plasmid vector. The vector was cotransfected into Sf9 cells together with aae-miR-989 mimic, NC mimic, or aae-miR-989 mimic with mutations in the seed region (Fig. 4A). While *GFP* transcript levels were significantly downregulated (*P = *0.0004) in aae-miR-989 mimic transfected cells, NC or the mutant mimic had no effect on the *GFP* transcript levels (Fig. 4F). A similar result was observed at the protein level when cell samples from the same experiment were run on a Western blot, where anti-GFP was used as a control, although mutant mimic had some effect on GFP protein levels (Fig. 4G and H).

**FIG 5:**
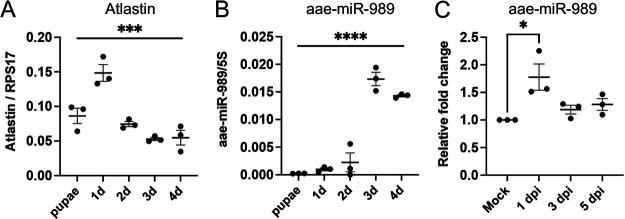
Negative correlation between the abundances of AaATL and aae-miR-989 in female mosquitoes. (A) Expression levels of AaATL in *Ae. aegypti* mosquitoes at the pupal stage and 1 to 4 days post emergence assessed by RT-qPCR analysis of RNA extracted from the samples. (B) Abundance of aae-miR-989 was assessed in samples in (A) to compare its abundance with transcript levels of AaATL. (C) Abundance of aae-miR-989 was assessed in samples in Fig. 1A to compare its abundance with transcript levels of AaATL. The error bars represent standard error of mean (SEM) of three biological replicates. One-way ANOVA test was carried out to determine statistical significance. *, *P < *0.05; ***, *P < *0.001; ****, *P < *0.0001.

Consistent with the results above, we found a negative correlation between the abundances of AaATL and aae-miR-989 in female mosquitoes from pupal stage to 4 days post adult emergence (Fig. 5A and B). Quantification of aae-miR-989 in samples from DENV-infected Aag2 cells (Fig. 1A) also showed a negative correlation between the miRNA and AaATL levels during DENV infection (Fig. 5C) and decrease in AaATL and increase in aae-miR-989 at 1 dpi followed by increases in AaATL levels from 2 to 5 dpi and reduced aae-miR-989 levels. Overall, the results suggested sequence-specific interaction of aae-miR-989 with AaATL target sequences and confirmed the negative regulation of AaATL by aae-miR-989.

## DISCUSSION

Flaviviruses, such as DENV, replicate their genomes in altered ER membranes due to the organelle’s versatile functions in cellular processes (25). Since ATLs in mammalian cells have been recognized as membrane fusogens that maintain the ER membrane structure and facilitate DENV replication in the cells, the involvement of ATL during DENV replication and *Wolbachia* transinfection in the mosquito *Ae. aegypti* was investigated since it has not yet been characterized in mosquitoes. The effect and cross-effect of *Wolbachia* and DENV infection on AaATL was also investigated to test the hypothesis that DENV replication is hindered upon *Wolbachia* infection because the expression of AaATL is downregulated, and AaATL has a significant involvement in flavivirus replication as seen in mammalian cells.

Flaviviruses use the ER membrane to establish their replication complexes (26). Atlastin enhances DENV replication by increasing the ER surface area. This is achieved by regulating ER tubule junctions by atlastin. Atlastins deficient cells lost their three-way ER tubule junctions (27). Further, in the characterization of human Atlastin 2, the GTPase activity was found to be important for DENV replication (17). Although human Atlastin 2 was found to interact with DENV NS3 protein, it is likely because of the regulation of ER tubule junctions by alastin. Both human Atlastin 2 and 3 affect DENV virion levels, but only Atlastin 2 reduces gRNA replication (17). Suppression of Atlastin 3 expression induced accumulation of immature DENV virions (17). Our results showed induction of AaATL following DENV infection in Aag2 cells and silencing the gene led to reduced DENV replication in Aag2 and C6/36 mosquito cells. These results suggest that similar to human atlastins, AaATL is a proviral factor for DENV. As there is only one atlastin in *Ae. aegypti*, it is possible that AaATL is involved in viral gRNA replication, virion assembly, and virion release. Future experimentation would still be ideal to further dissect the functional roles of AaATL, and this could involve examining virus protein localization. Therefore, an electron-microscopy analysis may increase understanding the function of AaATL.

miRNAs have been shown to play a role in mosquito-arbovirus interactions, with several miRNAs found differentially expressed upon virus infection (28). However, only a limited number of target genes of these differentially expressed miRNAs have been identified. In this study, a miRNA, aae-miR-989-3p, was found to suppress the expression of AaATL. The expression of aae-miR-989 was upregulated in Zika virus–infected *Ae. aegypti* and DENV-infected C6/36 cells (21, 23). However, the role of aae-miR-989 in DENV or Zika virus infection is unknown. In this investigation, oversupply of aae-miR-989 reduced DENV gRNA and virion levels. In addition, the target site of aae-miR-989 was found to be conserved between *Ae. aegypti* and *Ae. albopictus*, consistent with silencing *Ae. albopictus* atlastin expression leading to reduced DENV replication in C6/36 cells. Therefore, aae-miR-989 may play a role in host response to DENV infection by inhibiting AaATL expression, although there could be more direct targets of aae-miR-989 that could contribute to DENV replication.

*Wolbachia* is known to be associated with the ER and a recent study suggested a negative correlation between *Wolbachia* density and atlastin levels in *Drosophila* (29). In *Wolbachia* transinfected Aag2 cells, AaATL expression was upregulated in the case of Aag2.*w*AlbB and *w*MelPop cells; however, it is unknown whether this upregulation is caused by *Wolbachia*-induced ER remodeling (9). In *Ae. aegypti* mosquitoes transinfected with *w*AlbB, there was relatively less AaATL expression at 2 and 6 days post emergence but higher AaATL levels at 12 dpe compared to *Wolbachia*-free mosquitoes. Further, silencing AaATL in Aag2.*w*AlbB and *w*MelPop cells had no significant effect on *Wolbachia* density. The expression pattern of AaATL during *Wolbachia* or DENV infection did not support our hypothesis on AaATL being a factor in *Wolbachia*-mediated DENV inhibition.

Overall, the results showed that consistent with human atlastins, AaATL is a proviral protein during DENV replication in *Ae. aegypti* cells. Further, we found that AaATL is negatively regulated by aae-miR-989 miRNA. Our results also showed that AaATL does not seem to be required for maintaining *Wolbachia* density or contribute to *Wolbachia*-meditated DENV inhibition in mosquito cells. The results shed further light on the tripartite *Wolbachia*-DENV-mosquito interactions.

## MATERIALS AND METHODS

### Cells, mosquitoes, and virus infection.

The *Ae. aegypti* cell line (Aag2) persistently infected with *Wolbachia* strain *w*MelPop (Aag2.*w*MelPop) were used during *in vitro* experimentation and were grown in 1:1 Mitsushashi-Maramorosch and Schneider’s insect medium supplemented with 10% fetal bovine serum (FBS) at 27°C. In addition, Aag2.*w*AlbB cells, transinfected with purified *w*AlbB strain of *Wolbachia* from Aa23 cells, were used (30). Aag2, Aag2.*w*MelPop, and Aag2.*w*AlbB cells were infected at 1 MOI using ET300 DENV-2 stock prepared by amplification in C6/36 Aedes albopictus cells and diluted in 1:1 Mitsushashi-Maramorosch and Schneider’s medium containing 10% FBS. C6/36 cells were used for virus amplification because of an ineffective RNAi response during DENV replication due to a defective Dicer-2, which leads to higher virus yields (31). Mock-infected cells were used as control during the experiment and a 12-well plate was also prepared for infecting cells with medium containing 2% FBS. *w*AlbB-transinfected and tetracycline-cured *Ae. aegypti* mosquitoes (32) were used for analyzing AaATL expressions and RNA was extracted at 2, 6, and 12 days post-emergence (dpe).

### RNA extraction and RT-qPCR quantification.

Total RNA from cells and mosquito samples was extracted by using Qiazol, which was then DNase treated with TURBO DNase (Invitrogen). cDNA synthesis was performed by following the standard protocol of the M-MuLV reverse transcriptase kit (New England BioLabs) using specific primers for DENV-2 and oligo-dT primers for host genes (Table 1). A two-step qPCR was performed in duplicates using QuantiFast SYBR green PCR kit (Qiagen) and in a Qiagen Rotor-Gene Q under the following conditions: 95°C for 30s, and 40 cycles of 95°C for 10s and 60°C for 45s, followed by the melting curve (68°C to 95°C).

**TABLE 1:** Primers and siRNAs sequences used in this study

Primer name	Sequence (5′ and 3′)
Atlastin-qF	TGGACTCGGAACACAATCGG
Atlastin-qR	GTCCAGATCCTGCTCCAACC
*w*AlbB-wsp-qF	ATCTTTTATGGCTGGTGGTGCT
*w*AlbB-*wsp*-qR	GGAGTGATAGGCATATCTTCAAT
*w*MelPOP-*wsp*-qF	ATCTTTTATAGCTGGTGGTGGT
*w*MelPOP-*wsp*-qR	GGAGTGATAGGCATATCTTCAAT
DENV2-qF	GGTATGGTGGGCGCTACTA
DENV2-qR	CAAGGCTAACGCATCAGTCA
AeRPS17-qF	CACTCCGAGGTCCGTGGTAT
AeRPS17-qR	GGACACTTCGGGCACGTAGT
AaATL-siRNA 1^*a*^	UCUUCGUUCAGUGUGAAUGAG
AaATL-siRNA 2^*a*^	AUACUUCGAGUACAUAUACCG
NC-siRNA^*a*^	UUCUCCGAACGUGUCACGUTT
GFP-qF	CCCAAGCTTCGCCACCATGGTGAGCAA
GFP-qR	CGGGGTACCCTTGTACAGCTCGTCCATGC
ATL Target F	GTCTAGAGCAGTCCCTCCGACGTCACATCACCTCCCCGCGGG
ATL Target R	CCCGCGGGGAGGTGATGTGACGTCGGAGGGACTGCTCTAGAC
NC-mimic^*a*^	UUCUCCGAACGUGUCACGUTT

a siRNAs and miRNA mimics were duplex short RNAs. The complementary strands are not shown.

For quantification of aae-miR-989, small RNAs were first reverse transcribed with miScript II RT kit (Qiagen) using the HiSpec buffer with 250 ng of total RNA per sample according to the manufacturer’s instructions. Quantitative PCR was followed with a miScript SYBR green PCR kit (Qiagen) in a Qiagen Rotor-Gene Q using 10 times dilution of cDNA per reaction. For aae-miR-989, the miRNA sequence was used for the forward primer sequence while 5s rRNA was used as the normalizing small RNA.

RT-qPCR data were analyzed using the relative expression ratio method (Ratio = (*E*_target_)^ΔCP^_target(control – sample)_/(*E*_ref_)^ΔCP^_ref(control – sample)_) as described previously (33). Gene expression levels or DENV gRNA levels in controls were adjusted to 1 and the transcript levels in treatments are expressed as fold changes relative to the controls.

### Silencing AaATL.

The potential effect of AaATL on DENV replication in Aag2 cells and *Wolbachia* density in *Wolbachia*-infected cells was analyzed through RNA interference (RNAi)-mediated gene silencing of AaATL. Transfection in Aag2 cells was conducted with Cellfectin II (Invitrogen) and serum free transfection medium. Transfection was performed in 12-well plates in replicates, including Cellfectin II, a negative-control siRNA, or AaATL siRNA (synthesized by Genepharma) (Table 1). Cells were incubated overnight at 27°C and 24h after transfection they were infected with DENV at MOI 1. Cells and supernatants were collected at 2 and 5 days postinfection.

AaATL was also silenced in Aag2.*w*AlbB and Aag2.*w*MelPop cells to assess effect on *Wolbachia* density by using the same siRNA transfection procedure mentioned above. For *Wolbachia* density, genomic DNA was first extracted from the transfected cells through the DIY spin column protocol (34). A two-step qPCR with melt curve analysis for Aag2.*w*AlbB and Aag2.*w*MelPop DNA was performed as per the manufacturer’s instructions (Qiagen) using previously developed primers that targeted the *AeRPS17* gene and the *Wolbachia surface protein* gene (*wsp*) (Table 1).

### Focus forming assay for DENV-2 virion titration.

Through focus forming assay, DENV-2 virions were titrated using C6/36 *Ae. albopictus* cells using the procedure previously described (35). Seeded in a 96-well plate, C6/36 cells were inoculated with serially diluted medium (10^−1^, 10^−2^, 10^−3^, 10^−4^) collected from experiments. Plates were rocked for 1 h at room temperature and then incubated at 37°C for another hour. After incubation, the medium was removed, and cells were overlaid with 1.5% carboxymethyl cellulose (CMC) and 2.5% FBS in Opti-MEM medium. A following incubation period of 72 h at 27°C occurred before removing the overlay and fixing the cells for 20 min at −20°C with 80% ice-cold acetone in PBS before air drying overnight. PBST was used to block the cells at 37°C for 30 min before undergoing a 2 h of incubation period at 37°C with the primary DENV-2 envelope (human) antibody diluted (1:1000) with PBST. Plates were washed thrice with PBST and incubated for 1 h at 37°C with the secondary antibody (IRDye800CW goat anti-human LICOR). A second washing of the plates occurred, followed by a drying and scan on the Odyssey imager (LI-COR Biosciences) at 41 mM resolution to count foci and calculate viral titer, respectively. Focus forming units (FFUs) were obtained by performing titrations in triplicates.

### miRNA-target interaction.

To find potential target site(s), the full-length sequence of AaATL mRNA was used against all the known *Ae. aegypti* miRNAs available on miRBase. For this, we used RNA22 software (https://cm.jefferson.edu/rna22/Interactive/) given stringent criteria, including maximum complementarity in the seed region (nucleotides 2 to 8 from the miRNA 5′ end) as well as maximum folding energy for heteroduplex (Kcal/mol). aae-miR-989 showed the best folding energy (−22.10 Kcal/mol) and complete complementarity with the seed region.

To explore the interaction of aae-miR-989 with AaATL as well as effect on DENV replication, Aag2 cells were transfected with aae-miR-989 mimic and negative-control mimic (synthesized by Genepharma) 100 μM each, or Cellfectin II transfection reagent only. After 24 h of transfection, cells were infected with DENV at MOI 1 and collected at 5 dpi.

To assess the direct interaction of aae-miR-989 with AaATL target sequences, two oligonucleotides (42 nt each Forward and Reverse, Table 1) containing target sequence were designed with restriction sites of XbaI and SacII incorporated at the 5′ and 3′ ends, respectively. The sequence of reverse oligonucleotide was reverse complementary to the forward one. For annealing, both oligonucleotides (10 μM each) were mixed and annealed at 94°C for 5 min and slowly cooled down to room temperature for 45 min. The resulting dimer was digested with XbaI and SacII restriction enzymes, purified and cloned into the pIZ/GFP vector downstream of the GFP coding region. Clones were sequenced for confirmation. The plasmid (1 μg) and 100 μM mimic, control mimic, mutated mimic, or Cellfectin II transfection reagent only were cotransfected into Sf9 cells, which were grown in SF900 III medium (ThermoFisher Scientific). We used Sf9 cells as a heterologous cell line to assess the direct interaction of aae-miR-989 with AaATL target sequences. Heterologous cell lines are commonly used for miRNA-target interaction experiments with a reporter gene to avoid the effect of the endogenous miRNA being studied on the reporter construct. A miRBase (mirbase.org) search of Spodoptera frugiperda, from which Sf9 cells are derived, showed no match for aae-miR-989. Three days posttransfection cells were collected from which RNA was extracted and subjected to RT-qPCR using specific primers to *GFP* (Table 1) to assess the expression levels of *GFP*.

Protein samples from the same experiment as above were also assessed on a Western blot using an anti-GFP antibody (Invitrogen) as the primary antibody and an anti-rabbit (Sigma) antibody as the secondary antibody both at a 1:10,000 dilution. For reference protein, the same blot was hybridized with anti-Histone 3 (Life Technologies) followed by secondary anti-mouse antibody (Invitrogen) both at 1;10,000 dilutions. The blot was developed by using nitroblue tetrazolium chloride (NBT) and 5-bromo-4-chloro-3-indolylphosphate (BCIP) reagents. The density of GFP bands were semiquantified relative to those of the histone protein using ImageJ.
