# Supplementary material for: The Involvement of Atlastin in Dengue Virus and Wolbachia Infection in Aedes aegypti and Its Regulation by aae-miR-989
Source: Microbiol Spectr. 2022 Sep 27;10(5):e02258-22. doi: 10.1128/spectrum.02258-22 (PMC9603060; doi:10.1128/spectrum.02258-22)
Supplement: Supplemental file 1 — Supplemental material. Download spectrum.02258-22-s0001.pdf, PDF file, 4.6 MB [file spectrum.02258-22-s0001.pdf]

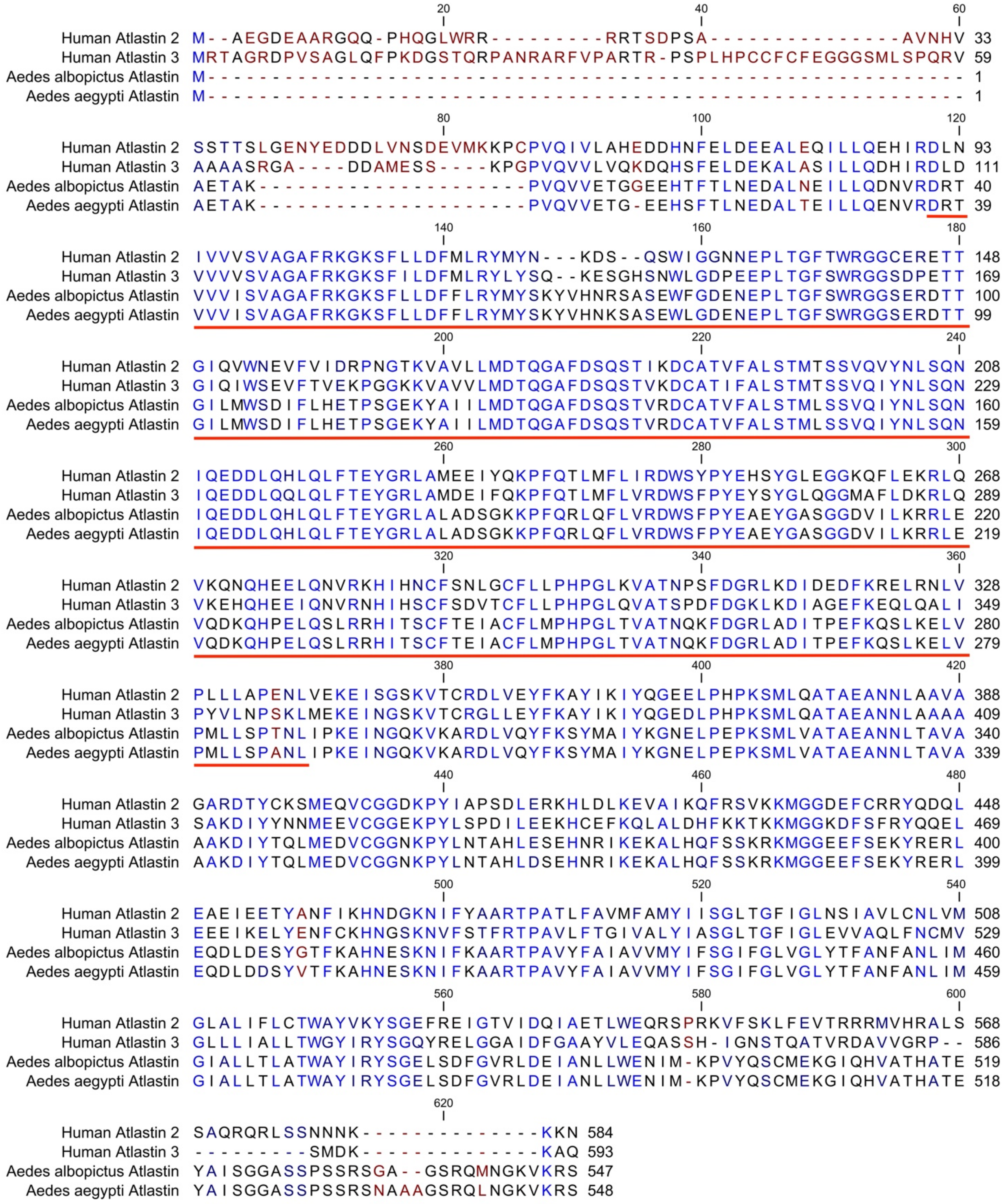

Figure S1: Amino acid sequence alignment of AaATL, *Ae. albopictus* ATL, and human ATLs. The red line under *Aedes aegypti* Atlastin indicates the guanylate-binding domain conserved in atlastins.

```
                                           20                     40                     60                     80
Aedes aegypti Atlastin   ATGGCAGAAA CGGCGAAACC GGTCCAGGTG GTCGAAACCG G---CGAGGA GCACTCATTC ACACTGAACG AAGATGCCCT 77
Aedes albopitus Atlastin ATGGCAGAAA CGGCGAAACC GGTCCAGGTG GTCGAAACCG GTGGCGAGGA ACACACATTC ACACTGAACG AAGATGCACT 80
                                          100                    120                    140                    160
Aedes aegypti Atlastin   GACGGAAATT TTGCTGCAGG AAAATGTGCG AGACCGAACG GTGGTTGTCA TTTCAGTTGC CGGAGCATTC CGGAAAGGCA 157
Aedes albopitus Atlastin GAACGAAATA CTGCTGCAGG ACAACGTGCG GGACCGGACG GTGGTGGTCA TCTCAGTTGC CGGGGCGTTC CGTAAAGGCA 160
                                          180                    200                    220                    240
Aedes aegypti Atlastin   AAAGTTTCTT GTTGGATTTC TTCCTGCGGT ATATGTACTC GAAGTATGTT CACAATAAAA GCGCCTCAGA ATGGCTGGGA 237
Aedes albopitus Atlastin AAAGCTTCCT GCTGGATTTC TTCCTGCGAT ATATGTACTC GAAGTACGTT CATAACAGAA GCGCCTCGGA ATGGTTTGGT 240
                                          260                    280                    300                    320
Aedes aegypti Atlastin   GATGAAAATG AACCCCTCAC AGGATTCTCG TGGCGGGGTG GCAGCGAACG GGACACAACC GGCATCCTAA TGTGGTCGGA 317
Aedes albopitus Atlastin GATGAGAATG AACCACTCAC AGGATTCTCG TGGCGGGGTG GCAGCGAGCG GGACACAACC GGCATCCTGA TGTGGTCCGA 320
                                          340                    360                    380                    400
Aedes aegypti Atlastin   CATATTTCTG CATGAGACAC CAAGTGGAGA AAAGTATGCC ATCATCCTGA TGGACACCCA GGGCGCCTTC GACAGTCAAA 397
Aedes albopitus Atlastin TATATTTTTG CATGAGACGC CAAGTGGAGA AAAGTACGCC ATCATCTTAA TGGACACCCA GGGCGCCTTC GACAGCCAGA 400
                                          420                    440                    460                    480
Aedes aegypti Atlastin   GCACAGTGCG GGATTGTGCC ACAGTGTTTG CTCTCAGCAC AATGCTCTCG TCCGTACAGA TATACAACCT GTCACAGAAC 477
Aedes albopitus Atlastin GCACAGTGCG AGACTGTGCC ACAGTGTTCG CCCTCAGCAC GATGCTCTCA TCCGTACAGA TCTACAACCT GTCGCAGAAC 480
                                          500                    520                    540                    560
Aedes aegypti Atlastin   ATTCAGGAAG ATGACCTCCA ACACCTGCAG CTTTTCACCG AGTACGGTAG GTTGGCGTTG GCCGACAGTG GCAAGAAACC 557
Aedes albopitus Atlastin ATCCAGGAAG ACGACCTCCA GCACCTGCAG CTTTTCACCG AATACGGTCG GTTGGCATTG GCCGACAGTG GCAAGAAACC 560
                                          580                    600                    620                    640
Aedes aegypti Atlastin   CTTCCAGCGG CTACAGTTTC TGGTCCGAGA CTGGAGCTTT CCGTACGAGG CTGAATACGG TGCATCTGGA GGCGACGTGA 637
Aedes albopitus Atlastin GTTCCAGCGG CTACAGTTCC TGGTTCGGGA CTGGAGCTTC CCATACGAAG CGGAATACGG TGCCTCAGGA GGCGATGTCA 640
                                          660                    680                    700                    720
Aedes aegypti Atlastin   TTCTGAAGCG GCGCTTGGAA GTGCAAGACA AACAACACCC CGAACTGCAG TCCCTCCGAC GTCACATCAC CTCCTGCTTC 717
Aedes albopitus Atlastin TTCTAAAACG GCGCCTGGAA GTGCAAGATA AACAGCATCC CGAACTGCAA TCCCTCCGAC GTCACATCAC CTCCTGCTTC 720
                                          740                    760                    780                    800
Aedes aegypti Atlastin   ACGGAAATCG CATGCTTTCT GATGCCGCAT CCAGGTCTGA CGGTGGCCAC TAACCAGAAG TTTGACGGCC GACTAGCAGA 797
Aedes albopitus Atlastin ACGGAAATTG CATGCTTTTT GATGCCCCAC CCTGGACTGA CGGTGGCCAC CAACCAGAAG TTCGATGGCC GACTAGCGGA 800
                                          820                    840                    860                    880
Aedes aegypti Atlastin   CATTACGCCC GAGTTCAAAC AGAGCCTGAA GGAACTGGTA CCGATGCTAC TGTCGCCGGC AAATCTGATA CCCAAGGAAA 877
Aedes albopitus Atlastin CATAACGCCC GAGTTCAAAC AAAGCCTGAA GGAACTGGTA CCGATGCTGC TGTCGCCGAC CAATCTGATA CCGAAGGAAA 880
                                          900                    920                    940                    960
Aedes aegypti Atlastin   TCAACGGACA GAAGGTGAAA GCACGCGACC TCGTTCAGTA CTTCAAGTCC TACATGGCCA TCTACAAGGG TAACGAACTG 957
Aedes albopitus Atlastin TCAACGGCCA GAAGGTGAAG GCCCGTGACC TGGTTCAGTA CTTCAAGTCC TACATGGCCA TCTACAAGGG AAACGAACTG 960
                                          980                  1,000                  1,020                  1,040
Aedes aegypti Atlastin   CCTGAGCCGA AGAGCATGCT GGTGGCGACG GCAGAGGCGA ACAATCTGAC CGCAGTGGCA GCCGCTAAGG ACATCTACAC 1037
Aedes albopitus Atlastin CCCGAACCGA AGAGCATGCT GGTGGCGACG GCAGAGGCGA ACAATTTGAC CGCGGTGGCA GCCGCCAAGG ACATCTACAC 1040
                                        1,060                  1,080                  1,100                  1,120
Aedes aegypti Atlastin   CCAGCTGATG GAGGATGTCT GCGGTGGAAA CAAACCGTAC TTGAACACGG CACATTTGGA CTCGGAACAC AATCGGATCA 1117
Aedes albopitus Atlastin CCAGCTGATG GAGGACGTTT GCGGTGGAAA CAAACCGTAT CTGAACACGG CACACCTGGA GTCGGAACAC AATCGGATCA 1120
                                        1,140                  1,160                  1,180                  1,200
Aedes aegypti Atlastin   AGGAGAAGGC CCTGCATCAA TTCTCCTCCA AGCGGAAGAT GGGCGGCGAA GAGTTCTCCG AAAAGTACCG CGAGCGGTTG 1197
Aedes albopitus Atlastin AGGAGAAGGC GCTGCATCAG TTCTCCTCCA AGCGGAAGAT GGGCGGCGAA GAGTTCTCCG AGAAGTACCG CGAGCGGTTG 1200
                                        1,220                  1,240                  1,260                  1,280
Aedes aegypti Atlastin   GAGCAGGATC TGGACGACTC GTACGTCACG TTCAAGGCGC ACAACGAGAG CAAAAACATT TTCAAGGCGG CCCGGACACC 1277
Aedes albopitus Atlastin GAGCAGGACC TGGACGAGTC GTACGGCACC TTCAAGGCGC ACAACGAGAG CAAAAACATC TTCAAGGCGG CCCGGACGCC 1280
                                        1,300                  1,320                  1,340                  1,360
Aedes aegypti Atlastin   GGCAGTCTAC TTTGCCATTG CCGTCGTCAT GTACATCTTC AGCGGTATCT TCGGTCTAGT TGGACTGTAC ACGTTTGCCA 1357
Aedes albopitus Atlastin GGCGGTCTAC TTTGCGATTG CCGTCGTCAT GTACATCTTC AGCGGTATCT TCGGGCTGGT CGGCCTGTAC ACGTTTGCTA 1360
                                        1,380                  1,400                  1,420                  1,440
Aedes aegypti Atlastin   ACTTTGCCAA CCTGATCATG GGCATCGCAC TGCTAACGTT GGCCACGTGG GCCTACATTA GATATAGCGG CGAACTGAGC 1437
Aedes albopitus Atlastin ACTTTGCCAA CCTGATCATG GGCATCGCAC TGTTGACGTT GGCCACCTGG GCGTATATTA GGTACAGCGG CGAACTGAGC 1440
                                        1,460                  1,480                  1,500                  1,520
Aedes aegypti Atlastin   GATTTTGGCG TGCGGTTAGA CGAAATTGCC AACCTGCTGT GGGAAAATAT CATGAAACCA GTGTATCAAT CGTGCATGGA 1517
Aedes albopitus Atlastin GACTTTGGCG TGCGGTTAGA CGAGATAGCC AACCTGCTGT GGGAAAATAT CATGAAACCG GTCTATCAAT CCTGCATGGA 1520
                                        1,540                  1,560                  1,580                  1,600
Aedes aegypti Atlastin   GAAGGGTATC CAGCATGTGG CAACCCACGC GACCGAGTAT GCCATCAGCG GCGGGGCGTC ATCTCCGTCG TCCCGGTCAA 1597
Aedes albopitus Atlastin GAAGGGTATC CAGCATGTGG CGACCCACGC GACCGAGTAC GCCATCAGCG GCGGAGCGTC ATCACCTTCG TCCCGGTC-- 1598
                                        1,620                  1,640
Aedes aegypti Atlastin   ATGCCGCTGC CGGTTCTCGT CAGCTGAACG GTAAGGTGAA GCGCTCTTGA 1647
Aedes albopitus Atlastin ----CGGCGC AGGTTCGCGC CAAATGAACG GTAAAGTGAA GCGCTCTTGA 1644
```

Figure S2: Nucleotide sequence alignment of AaATL and *Ae. albopictus* ATL. The AaATL siRNA 1 position is boxed red.
